# Real-time MR-guided transarterial aortic valve implantation (tavi): in vivo evaluation in swine

**DOI:** 10.1186/1532-429X-13-S1-O54

**Published:** 2011-02-02

**Authors:** Harald H Quick, Philipp Kahlert, Holger Eggebrecht, Gernot M Kaiser, Nina Parohl, Juliane Albert, Lena Schäfer, Ian McDougall, Raimund Erbel, Mark E Ladd

**Affiliations:** 1 Institute of Medical Physics, University of Erlangen, Erlangen, Germany; 2 Department of Cardiology, University Hospital Essen, Essen, Germany; 3 Department of Transplantation Surgery, University Hospital Essen, Essen, Germany; 4 Institute of Diagnostic and Interventional Radiology, University Hospital Essen, Essen, Germany; 5 Evasc Medical Systems, Vancouver, BC, Canada

## Objective

Transcatheter, transarterial aortic valve implantation (TAVI) is rapidly emerging as a promising new treatment option for patients with severe symptomatic aortic valve stenosis who are considered at high or prohibitive surgical risk. Envisioning real-time MR guidance of the TAVI procedure, the objective of this study was the systematical *in vitro* evaluation of the MR imaging characteristics of a currently commercially available TAVI prosthesis and its delivery device and subsequent modification of the delivery device towards MR-compatibility. Featuring the modified MR compatible stent valve delivery device, real-time MR guided TAVI has been performed *in vivo* in eight swine.

## Methods

The self-expandable Medtronic CoreValve® aortic bioprosthesis (Medtronic, Minneapolis, USA) is composed of a nitinol stent frame with an integrated trileaflet porcine pericardial tissue valve and is either implanted via the femoral or subclavian artery. Its delivery catheter has a 12Fr shaft with 18Fr distal end comprising the crimped prosthesis which can be released stepwise. The original catheter shaft revealed ferromagnetic attraction thus potentially compromizing MR imaging and safety. The delivery device consequently was re-designed obviating any metal braiding resulting in full MR compatibility of the delivery device. MR-guided TAVI was performed on 8 farm pigs (75-85 kg) via transfemoral (2/8) and via subclavian (6/8) access on a 1.5 T MRI system (Avanto, Siemens, Germany) equipped with an interventional in-room monitor. Catheter placement and stent release was performed under real-time MR-guidance with a rt-TrueFISP sequence providing 5 fps.

## Results

**Figure 1:**
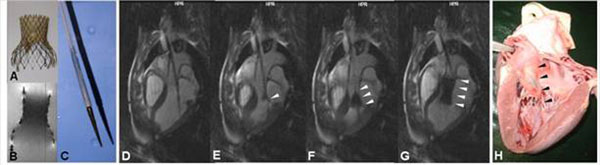
Nitinol CoreValve (A) featuring an aortic valve formed from porcine pericardial tissue, MR image (B); modified MR compatible delivery catheter (C). (D-G) Real-time TrueFISP images of MR-guided CoreValve deployment in vivo. Arrowheads in (E-G) show successive stent release and final stent position in histologic correlation (H).

The nitinol-based self-expandable stent-valve was excellently visualized with delineation even of small details. The commercial delivery catheter shaft of this device revealed strong ferromagnetic artifacts, thus also raising concerns regarding RF-related device heating and ferromagnetic attraction. Replacement of the commercial delivery device by the re-designed delivery device resulted in artifact elimination and excellent real-time visualization of catheter movement and valve deployment. MR-guided TAVI was successful in 6/8 swine (Figure 1). Post-interventional therapeutic success could be confirmed using ECG-triggered cine-TrueFISP sequences and flow-sensitive phase contrast sequences. Final stent valve position was confirmed by ex vivo histology.

## Conclusions

The self-expandable CoreValve aortic stent-valve is potentially suited for real-time MRI-guided placement after suggested design modifications of the delivery-system. MR imaging in this interventional setup provided excellent pre-interventional anatomic and functional evaluation of the native aortic valve, precise real-time instrument guidance allowing accurate placement of the stent-valve within the native aortic annulus, and finally detailed post-interventional evaluation of therapeutic success.

